# Statistical structure of lateral connections in the primary visual cortex

**DOI:** 10.1186/2042-1001-1-3

**Published:** 2011-01-26

**Authors:** Jonathan J Hunt, William H Bosking, Geoffrey J Goodhill

**Affiliations:** 1 Queensland Brain Institute and School of Mathematics and Physics, University of Queensland, St Lucia, QLD 4072, Australia; 2 Center for Perceptual Systems, 1 University Stations, #A8000, University of Texas, Austin, TX 78712, USA

## Abstract

**Background:**

The statistical structure of the visual world offers many useful clues for understanding how biological visual systems may understand natural scenes. One particularly important early process in visual object recognition is that of grouping together edges which belong to the same contour. The layout of edges in natural scenes have strong statistical structure. One such statistical property is that edges tend to lie on a common circle, and this 'co-circularity' can predict human performance at contour grouping. We therefore tested the hypothesis that long-range excitatory lateral connections in the primary visual cortex, which are believed to be involved in contour grouping, display a similar co-circular structure.

**Results:**

By analyzing data from tree shrews, where information on both lateral connectivity and the overall structure of the orientation map was available, we found a surprising diversity in the relevant statistical structure of the connections. In particular, the extent to which co-circularity was displayed varied significantly.

**Conclusions:**

Overall, these data suggest the intriguing possibility that V1 may contain both co-circular and anti-cocircular connections.

## Background

Grouping edges which belong to the same object or contour is a vital part of object recognition. Biological vision systems excel at this task, yet it is still extremely challenging for artificial vision systems [1]. In humans, contour detection develops well after birth [2], suggesting that learning from visual experience plays an important role.

The layout of edges in natural scenes has strong statistical structure. One well-known property is co-linearity. Co-linearity is important in contour grouping and texture discrimination tasks [3-5]. A more recently discovered generalization of co-linearity displayed by natural scenes is co-circularity: edges tend to be tangent to a common circle more often than would be expected by chance [6]. Furthermore, the degree of co-circularity in a contour can be used to predict human contour detection performance [7]. This raises the question of what biological substrate underlies this effect of co-circularity on performance.

An obvious candidate is the pattern of excitatory lateral connections in the primary visual cortex (V1). These connections are primarily found in layer 2/3 and are longer range than the inhibitory connections found predominately in other layers [8]. In humans these connections develop after birth, and their development coincides with improvement in contour detection [2,9,10]. Furthermore they have been shown to play a role in co-linear facilitation [11-14].

It has been previously demonstrated that long-range lateral connections connect preferentially to patches of similar orientation and primarily along the axis of orientation in V1 [15-18] (although others have claimed this preference is weak [8]). This is consistent with their role in co-linear facilitation. Although previous work has demonstrated that the variance in some of these data is not adequately explained by co-linearity alone [19], whether there is a more general tendency towards co-circularity in these connections has not been investigated.

Here we reanalyzed previously published functional anatomical data regarding lateral connections in tree-shrew [17], using a noise-resistant measure of co-circularity that we recently introduced to study the structure of orientation maps [20]. Surprisingly, we found a large variation in the statistics of lateral connections between animals. One unifying explanation for both this and previous results is that lateral connections in V1 are connected in a variety of ways, both co-circularly and anti-cocircularly. We suggest reasons why this may be a desirable arrangement for analyzing the structure of visual scenes.

## Methods

We reanalyzed data from four tree shrews previously published by Bosking *et al*. [17]. The experimental methods used are described in detail in that paper and we provide only a brief overview here. Tree shrew orientation preference maps were obtained using optical imaging. Additionally, 540 nm light was used to map surface blood vessels used for alignment. Biocytin was then injected into a specific site in V1 and the animal was sacrificed 16 hours later. Slices of V1 were imaged to locate the biocytin bouton and the surface blood vessels. The blood vessel information was then used to align the orientation preference maps with the bouton images giving overlaid information on the underlying connectivity from the injection site on the animal. The original experiment used a total of ten cases, however, we were only able to recover the data for four cases.

### Topography

**Figure 1:**
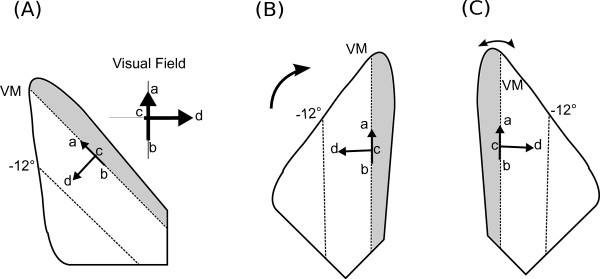
**Topography matching of tree shrew maps**. **(A) **shows a schematic of the topography of the tree shrew. The area shaded grey is a compressed topography of the ipsilateral hemisphere and is excluded from analysis due to its very different magnification factor. To facilitate analysis the maps are rotated to align the vertical axis **(B) **and then flipped to give a right-handed co-ordinate system **(C)**.

In order to accurately quantify the co-circularity present in these results it is important to know the underlying topography. Fortunately, the topography of the tree-shrew is consistent between individuals and well characterised [21]. To align the maps (and overlaid boutons) with visual space they were first rotated so the V1/V2 border was vertical, then flipped along the vertical axis to give a right-handed co-ordinate system (shown schematically in Figure 1). The tree-shrew V1 has a compressed representation of the ipsilateral visual field near the V1/V2 border with a very different magnification factor from the rest of V1. We therefore drew a line representing the edge of the contralateral visual field (the vertical meridian) and eliminated the ipsilateral portion of the map. The map was then rotated slightly to ensure the vertical meridian was represented vertically.

**Figure 2:**
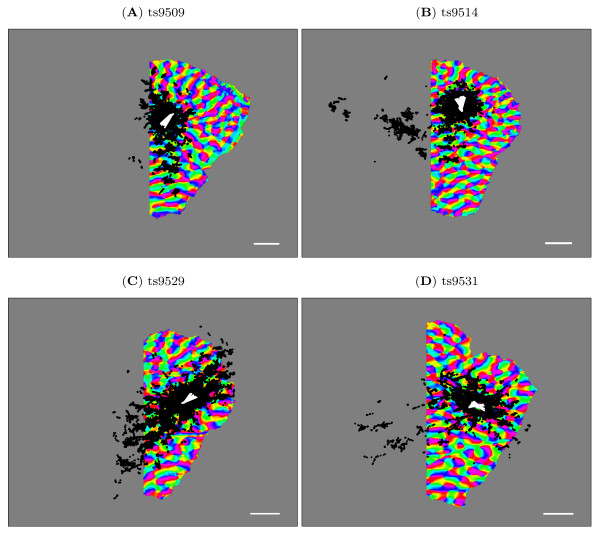
**Orientation preference maps with connections**. The orientation preference map for all cases with the injection site (white) and bouton traces (black) overlaid. The direction of the white injection sites indicates the preferred orientation at these points. The boutons indicate horizontal connections originating at the injection sites and connecting to the bouton sites. The orientation preference maps have been rotated, flipped and truncated (see methods) to create right-handed co-ordinate systems aligned with visual space and the overlaid boutons and injection sites have been similarly transformed after alignment. Scale bar is 1 mm.

This gave us an orientation preference map of V1 with an aligned injection site and bouton sites which indicate the lateral connections originating from the injection site. The rotating and flipping ensured that points on the map corresponded to points in visual space within a scaling factor. After truncation of the ipsilateral hemisphere the magnification factor was approximately constant for the region of V1 imaged, and cortical distance and direction could be used as a reliable proxy for visual field distance and direction. The aligned maps with overlaid bouton and injection sites are shown in Figure 2.

For the original analysis [17] electrophysiological recordings taken during the injection were used to determine orientation. We determined the orientation of the injection site using the orientation value of the underlying pixels that were measured using optical imaging. This differed from the electrophysiological recordings by an average of 15° (range 0-23°) from the optical orientation value (averaged across the animals).

### Characterizing the lateral connection statistics

**Figure 3:**
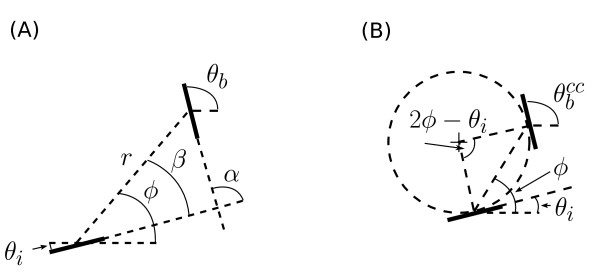
**Definition of terms**. **(A) **is a diagram of the various definitions of orientation and position between the injection site and the bouton site used in the methods. **(B) **shows a representation of eq. 1 showing co-circularity. Two edges are co-circular when they lie tangent to a common circle. Given an injection site with orientation  there is a unique orientation preference  (for any *ϕ*) that is exactly co-circular with the injection site.

To test for the presence of co-circularity in the lateral connections we adapted a noise-resistant measure of co-circularity introduced in Hunt *et al*. [20]. For each pair of injection/bouton sites on the orientation preference maps we defined the following (also shown diagrammatically in Figure 3A):

$$\begin{array}{ll} \theta_{i} & \text{orientation preference of injection site }i;\\ \theta_{b} & \text{orientation preference of bouton site }b;\\ r & \text{cortical distance between injection site and bouton site};\\ \phi & \text{angle of the line from injection site }i\;\text{to bouton site }b;\\ \alpha =\theta_{b}-\theta_{i}\;(\mathrm{mod}\;180{}^{\circ}) & \text{relative orientation preference of the bouton }b\;\text{relative to injection site }i;\\ \beta =\phi -\theta_{i}\;(\mathrm{mod}\;180{}^{\circ}) & \text{angular position of the bouton site }b\;\text{relative to the orientation preference}\\ & \text{of the injection site }i. \end{array}$$

The orientation preferences  and  are defined in real space where the stimulus is presented, relative to zero degrees along a horizontal *x*-axis. They are always taken on the *y*-positive side of the horizontal and thus range from 0° to 180°. The relative position of the cortical points *ϕ *is defined relative to the horizontal axis of the retinotopic map and is calculated modulo 180°. Except where noted, calculations of orientations and angular positions were done using modulo 180° arithmetic.

The wavelength *λ *of each map was calculated as the mean of the Fourier spectrum averaged over all directions as previously described [22]. As there is variation in column spacing between individuals [23], and the imaging of each map may be at different magnifications, the use of *λ *as the unit of length aims to ensure that the quantification of reduced symmetry is not affected by overall changes in map scaling. The wavelength gives a rough measure of the size of iso-orientation patches in the map.

We calculated a probability distribution for the connection statistics for each animal *p*(*α*, *β*, *r*). However, if *ϕ* is not uniformly distributed this can introduce spurious correlations. We corrected for this by calculating a four-dimensional probability *p*(, *β*, *r*, *ϕ*) and then marginalized along *ϕ*. This was done by discretizing both distance *r *and orientations , *β *and *ϕ *and binning the connections. Given the full distribution it was then possible to calculate the relevant marginal distributions such as *p*(|*β, r*) and *p*(*β*|, *r*).

### Co-circularity

If an injection site has orientation preference , then at a bouton site, located at angle *ϕ *in the cortex relative to the injection point, there is a unique orientation preference  that is fully co-circular with the injection site (Figure 3B):

(1) $$\theta_{b}^{\text{cc}}=2\varphi -\theta_{i}\left(\text{mod}\;180{}^{\circ}\right).$$

The difference in orientation between the actual orientation preference at the injection site , and the co-circular orientation , is a measure of the degree to which the bouton connection is co-circular. We define

(2) $$d_{\text{diff}}=|\theta_{b}-\theta_{b}^{\text{cc}}{|}_{\text{mod}180{}^{\circ}}\text{​}.$$

We use the notation |·|_mod 180° _to indicate that the absolute difference is calculated modulo 180°:

(3) $$|a{|}_{\text{mod}\;180{}^{\circ}}=\min\left(a\left(\text{mod}\;180{}^{\circ}\right),\;\left(-a\right)\left(\text{mod}\;180{}^{\circ}\right)\right).$$

Geometrically, this means that when calculating the co-circularity of an edge we always consider the smallest angle between the edge and the fully co-circular orientation . It follows that edges are never separated in orientation by more than 90°(for instance, if  = 135° and  then *d*_diff _= 65) so the maximum possible value of *d*_diff _is 90°. We calculated the mean *d*_diff _between injection sites and bouton sites at distance *r*:

(4) $$D_{\text{diff}}\left(r\right)\;\;=\;\;\;\frac{\sum_{\text{pairs}}|\theta_{b}-\theta_{b}^{\text{cc}}{|}_{\text{mod}\;180{}^{\circ}}}{N_{\text{pairs}}}$$

(5) $$=\;\;\frac{\sum_{\text{pairs}}|\;\theta_{i}+\theta_{b}-2\varphi {|}_{\text{mod}\;180{}^{\circ}}}{N_{\text{pairs}}}.$$

*D*_diff_(*r*) has the intuitively appealing property that it is 45° when no co-circularity is present (that is, when the connections from the injection site have no systematic dependence with orientation and angle) and decreases if the connections are connected co-circularly. To calculate *D*_diff_(*r*) we binned *r *in intervals of 1.5λ. The first bin, referred to hereafter as *r *= 0, contained pairs where 0 *≤ r <*0.75λ, the *r *= 1.5λ bin contained the pairs where 0.75λ *≤ r <*2.25λ and so on. This bin spacing balances the requirements of having a reasonable number of pairs in each bin to reduce noise, while allowing fine enough resolution to investigate trends with *r*.

In addition, the distribution of co-circularity from each injection/bouton pair can provide useful information since various distributions of *d*_diff_, the co-circularity of individual bouton sites (see eq. 2), would result in the same mean *D*_diff_(*r*). In addition to calculating the average co-circularity *D*_diff_(*r*), the values of *d*_diff _= |> +  − 2*ϕ*| _mod 180_° for each pair at distance *r *were therefore binned to create a histogram *H *(*r, d*). The *r *bins were the same as used for calculating *D*_diff_(*r*) and the *d*_diff _bins were spaced 15° apart. Since we were not interested in the absolute number of points at different distances, we calculated and plotted the conditional probability *P*(*d|r*). This allows a basic characterization of the distribution of co-circularity values.

### Co-circularity with offset

A more general property than co-circularity is co-circularity with offset . Co-circularity with offset > would arise if on a co-circular arrangement of edges, position was rotated independently of orientation. If lateral connections are co-circular due to co-circularity of natural scenes the maximal co-circularity strength should be found at  = 0. To test this we also calculated co-circularity with offset using the definition:

(6) $$D_{\text{diff}}(r,\;\tau )=\frac{\sum_{\text{pairs}}|\;\theta_{i}+\theta_{b}-2\varphi -2\tau {|}_{\text{mod}\;180{}^{\circ}}}{N_{\text{pairs}}}$$

for  = 0 to 80° in 10° increments. Since co-circularity is invariant under *ϕ → ϕ *+ 90° searching over this range covers all possible values for . We also calculated (*r*) for each value of *r*, which was defined as the value of  which minimized *D*_diff_(*r*, ).

### Testing for co-circularity

In the original analysis of the tree shrew data it was demonstrated that lateral connections are denser along the axis of injection site orientation and connect preferentially to sites of similar orientation. This evidence demonstrates that lateral connections are likely to prefer co-linearity. In order to test whether the co-circularity we found in the horizontal connections was simply due to this co-linearity, we recalculated *D*_diff_(*r*) (eq. 4), but excluded all points lying near the axis of the injection site. This was done by excluding all injection/bouton pairs with *β <*30° or *β >*150^°^.

### Controls and statistics

It was important to have a reliable control to assess the significance of any co-circularity measured in the maps. We are interested in the significance of any deviation from 45° that occurs in *D*_diff_(*r*). One comparison was simply examining the values of *D*_diff_(*r*, ) at  ≠ 0 since these are expected by our hypothesis to be less co-circular then with the true origin ( = 0). This means that we would expect  to be near 0 when connections are the most co-circular.

We created 99 control cases (*N *= 99) for each animal by adding Gaussian noise (*σ *= 100 pixels) to the bouton positions and calculated *D*_diff_(*r*)*_j _*for each of these controls (*j *denotes the index of the control). The added noise is high enough to overwhelm any co-circular preferences in the connections since it is much larger than the wavelength of the maps, which means that it is larger than the size of iso-orientation patches in the maps.

We then used a permutation test to find if the value *D*_diff_(*r*) for each animal differed significantly from the control values. We tested *D*_diff_(*r*) independently at each value of *r*. Permutation tests were used because we did not know what distribution the control values might be from and because we only had one true value to compare with the control distribution. We calculated the difference between the mean of the control values *D*_diff_(*r*)*_j _*and the animal case *D*_diff_(*r*):

(7) $$\Delta Q\left(r\right)\;=\;\frac{\sum_{j}D_{\text{diff}}{\left(r\right)}_{j}}{N}-D_{\text{diff}}(r).$$

Our null hypothesis was that *D*_diff_(*r*) was from the same distribution as the controls *D*_diff_(*r*)*_j _*. If the null hypothesis is true then the value of *D*_diff_(*r*) can be exchanged with one of the controls *D*_diff_(*r*)*_j _*without affecting the expected value of Δ*Q*(*r*). There are *N *+ 1 = 100 possible permutations under these exchanges (since we also include the original permutation). We calculated Δ*Q*(*r*)*_i _*, the value of Δ*Q*(*r*) for each of these permutations; using *i *as an index over all the permutations. We then calculated the significance of the true Δ*Q*(*r*) as the likelihood of this difference arising under the null hypothesis:

(8) $$p\left(r\right)=\frac{\sum_{i=1}^{N+1}H\left(\left|\Delta Q{\left(r\right)}_{i}\text{​}|-|\Delta Q\left(r\right)\right|\right)}{N+1}$$

where *H *(.) is the Heaviside function (*H *(*x*) = 0 for *x <*0, *H *(*x*) = 1 for *x ≥ *0). We considered *P <*0.05 to be significant.

### Natural scenes

We used an existing image dataset [24] for all natural scene analysis. These images are uncompressed, deblurred and have linearized intensity values and thus should have no artifacts due to compression or other steps in the image acquisition. This image set was also used when co-circularity in natural scenes was originally demonstrated [6]. The images are 12-bit greyscale with a resolution of 1536 *× *1024 pixels and an angular resolution of approximately 1 min of arc per pixel. The orientation and orientation strength of each pixel were calculated using steerable filters provided by matlabPyrTools [25]. After extracting orientation and orientation strength we calculated *D*_diff_(*r*) for each image analogously to how it was calculated for lateral connections. The exception was that when averaging *d*_diff _we weighted by the orientation strength of the pixels in the pair and we excluded pixels with very low orientation strength (essentially un-orientated pixels). This was done differently because we did not have information on orientation selectivity for most the animal data. We then calculated a natural scene value by re-averaging the values of *D*_diff_(*r*) for each scene to create an average across all scenes. We did this double averaging to ensure that the calculation was not overly preferenced towards particular types of scenes which have more orientation density (such as tree scenes).

## Results

### Alignment and topography

Figure 2 shows the aligned orientation preference maps with overlaid bouton and injection sites for all animals. In each case the vertical meridian was selected using previously published results of tree shrew topography [21] and the map rotated to make it vertical. All pixels representing receptive field positions in the ipsilateral visual field (that is, those to the right of the vertical meridian) were removed and these sections were not used in subsequent analysis due to the very different magnification factor in this region of the cortex. For analysis, the remaining region of V1 was treated as isotropic with a constant magnification factor. We have previously demonstrated that this approximation does not significantly affect our measures of co-circularity [20].

There was a varying number of boutons sampled from each animal. Boutons positioned outside the V1 orientation map or on the excluded side of the vertical meridian were ignored. The number of included boutons for each animal is listed in Table 1. Although there was a large number of boutons for each case, each bouton cannot be considered an independent measurement as many boutons are clustered together near a single region of the map.

**Table 1:** Number of included boutons for each animal.

Animal	**No**.
ts9509	5411
ts9514	4974
ts9529	10708
ts9531	4821

### Co-circularity

**Figure 4:**
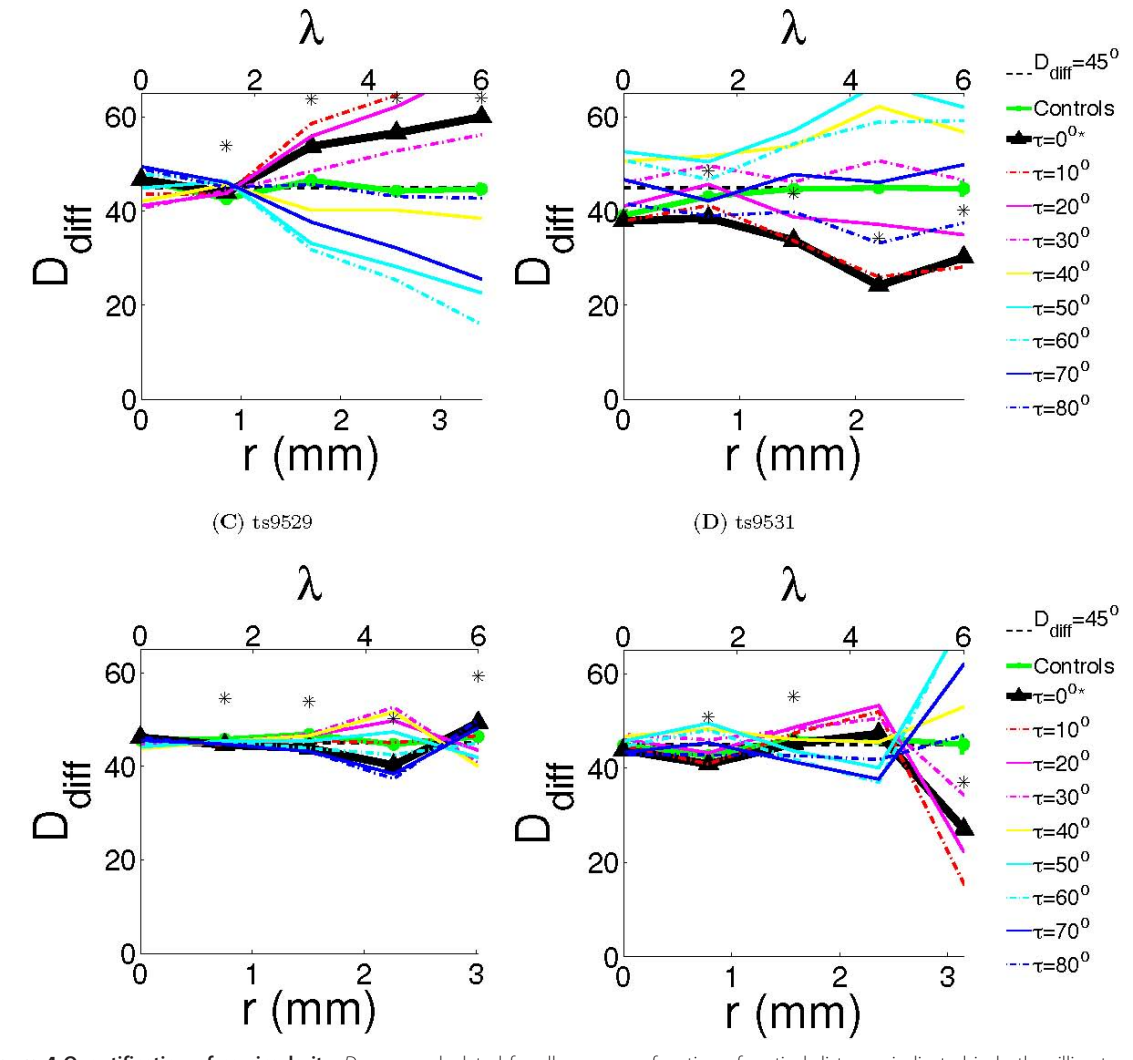
**Quantification of co-circularity**. *D*_diff _was calculated for all cases as a function of cortical distance, indicated in both millimeters and wavelengths *λ*. The dotted black line marks *D*_diff_(*r*) = 45°, deviations below this line indicate co-circularity. Significance (*P ≤ *0.05) compared with the control cases is marked as '*' on figure. We also report (*r*) and the difference *D*_diff_(*r*, 0) *− D*_diff_(*r*, (*r*)). **(A) **Case ts9509 had *D*_diff _significantly different from the control case at *r *= 1.5, 3, 4.5, 6λ (*P <*0.01). The values of *τ* which minimized the *D*_diff _at *r *= 1.5, 3, 4.5, 6λ were 10°, 60°, 60°, 60° respectively, with differences *D*_diff_(*r*, 0) − *D*_diff_(*r*, (*r*)) of 0.2°, 21.9°, 31.2°, 44.0° respectively. **(B) **Case ts9514 had *D*_diff _significantly different from the control case at *r *= 1.5, 3, 4.5, 6λ (*P <*0.01). The values of *τ* which minimized the *D*_diff _at *r *= 1.5, 3, 4.5, 6λ were 0°, 10°, 0°, 10° respectively, with differences *D*_diff_(*r*, 0) *− D*_diff_(*r*, >(*r*)) of 0°, 0.08°, 0°, 2° respectively. **(C) **Case ts9529 had *D*_diff _significantly different from the control case at *r *= 1.5, 3, 4.5, 6λ (*P <*0.01). The values of *τ* which minimized the *D*_diff _at *r *= 1.5, 3, 4.5, 6λ were 0°, 80°, 80°, 40° respectively, with differences *D*_diff_(*r*, 0) *− D*_diff_(*r*, (*r*)) of 0°, 0.7°, 2.9°, 9.3° respectively. **(D) **Case ts9531 had *D*_diff _significantly different from the control case at *r *= 1.5, 3, 6λ (*P <*0.04). The values of *τ* which minimized the *D*_diff _at *r *= 1.5, 3, 4.5, 6λ were 0°, 70°, 60°, 10° respectively, with differences *D*_diff_(*r*, 0) *− D*_diff_(*r*, (*r*)) of 0°, 3.9°, 10.3°, 11.5° respectively.

We quantified the co-circularity of the lateral connections for each animal separately. Figure 4 shows the calculated *D*_diff_(*r*, ) (see Methods) for each animal using the connections shown in Figure 2. *D*_diff _is a measure of co-circularity, values below 45° indicate co-circularity. Each animal was treated separately because there were substantial differences between individuals. To establish whether variations in *D*_diff _were significant, each animal was compared with 100 control cases generated by adding Gaussian random noise (*σ *= 100 pixels ≈ 1.5 mm) to the bouton positions. The noise was much larger than the wavelength of the maps (approximately 35 pixels), ensuring that boutons were moved significantly outside their original iso-orientation patch. Comparisons were made using nonparametric statistics (see Methods).

Co-circularity with offset  is a generalization of co-circularity. Edges are co-circular when tangent to a common circle and co-circularity with offset  is when edges occur at an orientation  to the tangent of a common circle (so  = 0 is co-circularity). Co-circularity with non-zero  is not found in natural scenes and thus we would predict that if co-circularity in the lateral connections is significant, then it should be present more strongly than co-circularity with any non-zero offset >. We measured co-circularity with offset at a range of non-zero offsets to test this hypothesis (Figure 4).

**Figure 5:**
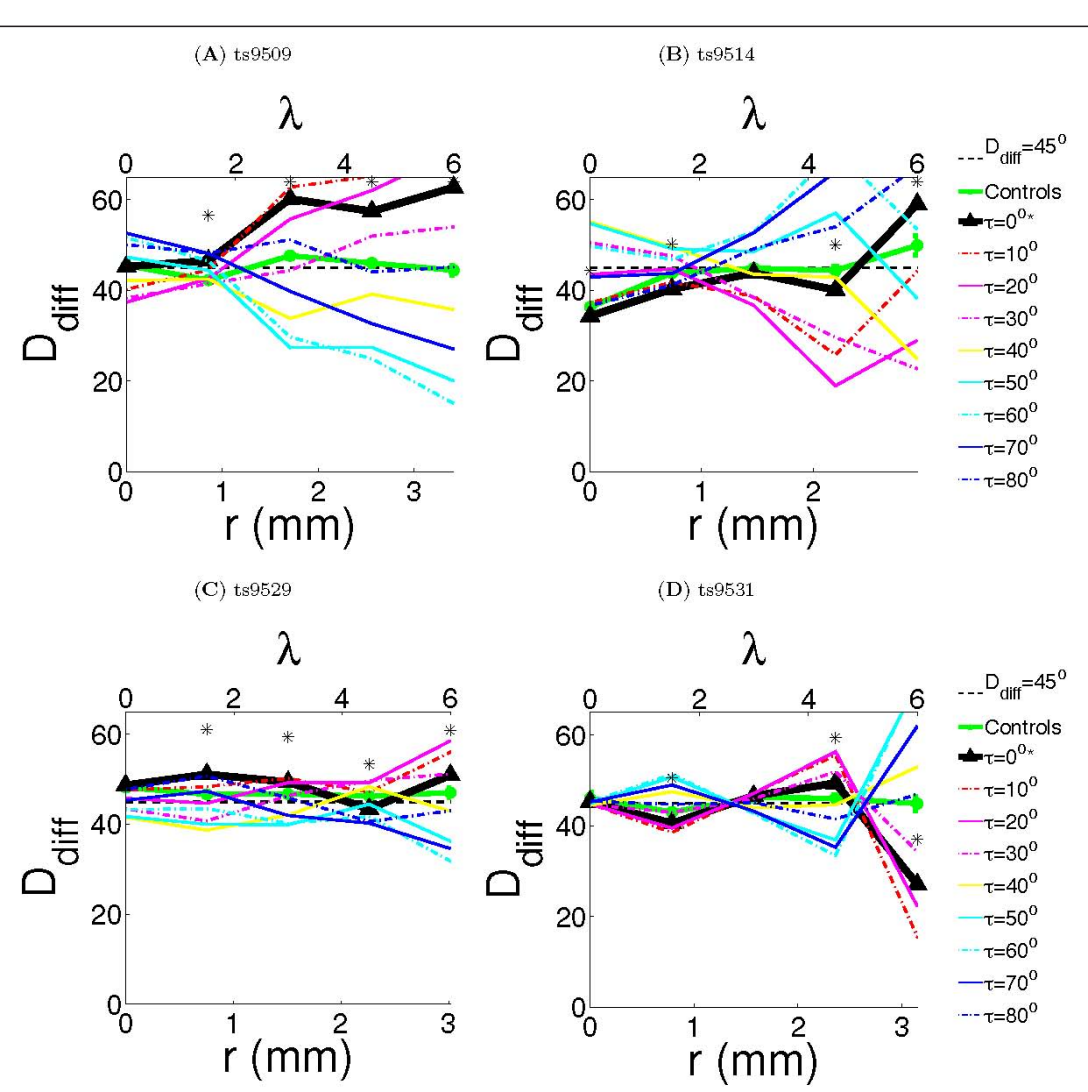
**Quantification of co-circularity excluding co-linearity**. *D*_diff_(*r*) was calculated with all bouton sites which are co-linear with the injection site excluded (30° *≥ β ≤ *150°). All cases where the true value was significantly different from controls were marked as '*'. (**A**) Case ts9509 had *D*_diff _significantly different from the control case at *r *= 1.5, 3, 4.5, 6λ (*P <*0.01). The values of *τ* which minimized the *D*_diff _at *r *= 1.5, 3, 4.5, 6λ were 30°, 50°, 60°, 60° respectively, with differences *D*_diff_(*r*, 0) − *D*_diff_(*r*, (*r*)) of 5°, 32.8°, 32.5°, 47.7° respectively. **(B) **Case ts9514 had *D*_diff _significantly different from the control case at *r *= 0, 1.5, 4.5, 6λ (*P <*0.02). The values of *τ* which minimized the *D*_diff _at *r *= 1.5, 3, 4.5, 6λ were 0°, 20°, 20°, 30° respectively, with differences *D*_diff_(*r*, 0) − *D*_diff_(*r*, >(*r*)) of 0°, 7.1°, 21.1°, 36.4° respectively. **(C) **Case ts9529 had *D*_diff _significantly different from the control case at *r *= 1.5, 3, 4.5λ (*P <*0.01). The values of *τ* which minimized the *D*_diff _at *r *= 1.5, 3, 4.5, 6λ were 40°, 50°, 70°, 60° respectively, with differences *D*_diff_(*r*, 0) *− D*_diff_(*r*, (*r*)) of 12.4°, 9.5°, 3.1°, 19.1° respectively. **(D) **Case ts9531 had *D*_diff _significantly different from the control case at *r *= 1.5, 4.5, 6λ (*P <*0.02). The values of *τ* which minimized the *D*_diff _at *r *= 1.5, 3, 4.5, 6λ were 10°, 60°, 60°, 10° respectively, with differences *D*_diff_(*r*, 0) − *D*_diff_(*r*, (*r*)) of 2.0°, 3.9°, 16.0°, 11.5° respectively.

These results indicate that the degree of co-circularity varies significantly between animals. However, previous work has demonstrated that lateral connections are elongated along their visual axis, indicating likely co-linearity [17]. Since co-circularity is a generalization of co-linearity, any co-linearity present contributes significantly to the degree of co-circularity. In order to clearly establish the presence of co-circularity rather than just that of co-linearity in the lateral connection preferences, we re-calculated our results with all co-linear connections excluded. Figure 5 shows the same measurements as the previous figure, but without co-linear connections.

Case ts9509 is strongly anti-cocircular at longer ranges (Figure 4) and this tendency remains even if co-linear connections are removed (Figure 5). Case ts9514 is strongly co-circular, although when co-linearity is excluded this tendency becomes less strong (and it is anti-cocircular at large *r*). The two other cases show inconsistent results at different wavelengths, although case ts9531 shows strong co-circularity for the longest connections.

Examining the results in the four cases at different values of  confirms that there is significant variation in the statistics of wiring between the four animals. Case 9509 has values of (*r*) which are near 45°, while case 9514 shows (*r*) near 0° (the other two cases show inconclusive results). These results demonstrate that the variation between co-circularity and anti-cocircularity between the cases is significant and are an additional verification that the wiring on these connections is not simply co-circular as our initial hypothesis predicted. As we discuss later, these findings indicate that the results cannot be explained by minor variations in topographical alignment between the animals.

**Figure 6:**
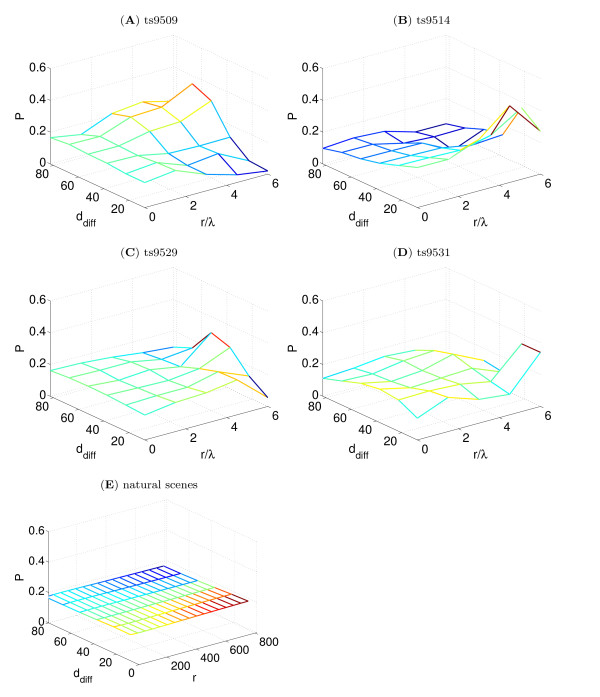
**Distribution of *d*_diff _values for the four cases compared with natural scene statistics**. **(A-D) **For each animal the probability distribution *P *(*d*_diff_|*r*) was calculated by binning into bins of 20° in *d*_diff _and 1.5λ in *r*. Because of the low number of connections the results have a significant amount of noise. **(E) **An analogous statistic was calculated for natural scenes. Although difficult to see at this scale (which matches that in panels A-D), there is small decline in probability with increasing *d*_diff _so the mean value *D*_diff_(*r*)* <*45°. Because a large number of natural scenes could be counted and averaged there was very little noise in these results.

In order to better understand the statistics of edge arrangements we also considered some other ways of examining edge statistics. Firstly, because multiple *d*_diff _distributions would result in the same mean value, we plotted the distributions of *d*_diff _and compared the distributions with those found in natural scenes (Figure 6). We found that in natural scenes the distribution of *d*_diff _(averaged across about 4,000 natural scenes) has a small constant negative gradient. Although the slope of this distribution is small, this represents a sample across many scenes and within an small region of a scene steeper gradients are often found. For the lateral connections the distributions appeared to be symmetric distributions centred (by definition) on the mean, *D*_diff_. This indicates that for each individual injection site the connectivity at a given wavelength tends to be centered on a particular value of co-circularity (that is, being either predominately co-circular or anti-cocircular or neutral).

Additionally, we directly examined the probability distribution of edges in both natural scenes and lateral connections (data not shown). We found connections are isotropic for short-range connections (as also seen in the *D*_diff _results). Long-range connections have strong biases for both orientation and direction. However, these statistics are probably due primarily to the previously studied elongation along the axis of the injection site orientation [17], rather than any specific arrangement of edges, as they are also present in controls (where noise is added to the bouton positions).

## Discussion

We examined lateral connections in V1 and compared the statistics of their connections with the edge arrangement of natural scenes. These connections develop after eye-opening [2,9] and therefore may be driven by natural scene input. As found previously, at short distances the lateral connectivity is isotropic (and probably mostly inhibitory [8]). However, long-range lateral connections are clearly anisotropic. We hypothesised that these connections might display similar connectivity statistics to edges in natural scenes. However, we found that the connectivity statistics showed a range of behaviour for the different individuals and both co-circularity and anti-cocircularity were present. This study is, as far as we know, the first to directly examine lateral connectivity in V1 for evidence of co-circular connections.

Some limitations of our results are as follows. Firstly, due to archiving problems, the original data from the study of [17] was only available in fragmentary form and we were only able to reconstruct sufficient information for our analysis from four tree shrews. Secondly, the experimental data has a significant amount of noise due to error in alignment of optical imaging with histology. Because co-circularity is a second order property these errors are compounded when calculating co-circularity statistics. An additional possible source of noise is based on more theoretical considerations and inherent to any experimental study of these connections. Suppose the brain has some desired function for specifying lateral connectivity (for instance some spread of co-circularity), it is unlikely this function is realised without a significant level of noise in the connectivity. Because of this, even without any experimental uncertainty, it may be difficult to recover a good approximation to the original generating function. Assuming the noise in connectivity is independent between coloumns, this can be overcome by increasing the number of injection sites, however, in this study only four sites could be used. The restricted captive environment in which the tree shrews were raised was probably not as rich as a truly natural environment, however, at the level of statistical characterisation considered here these differences are not likely to be consequential. Because of these limitations, it is difficult to make definitive conclusions from our results. However, this data is still able to provide some important insights into functional connectivity in V1.

The variations between the four animals are not explainable by minor variations in topography. We examined the value of co-circularity for each of the animals for various values of , which is the value of co-circularity that would be present if the assigned topography was rotated by . In order for, for instance, case ts9509 to be in agreement with case ts9514 they would have to contain a combined error of 45° in the assigned orientation of the topography. Other work has demonstrated that the topography of tree shrews has little inter-individual variability and consistent local structure [26]. Additionally, our previous work has shown that the co-circularity statistics we used are robust to other deviations in topography such as anisotropy.

Previous work [19] has shown that co-linearity alone does not fully explain the anisotropy in the original paper of Bosking *et al*. [17] and some broader form of connectivity is required. Here, we postulated that co-circularity similar to that found in natural scenes would explain this. However, we found that there is a large variation in co-circularity statistics between different injections sites (and animals). In particular case ts9509 shows strong anti-cocircularity, while case ts9514 shows strong co-circularity.

Several other studies have examined horizontal connections in V1 in a variety of animals. Early work in tree shrew [27] found long-range (around 2.5 mm) lateral connectivity and some indications of orientation selectivity in the connections, as did similar work in cats [28]. There is conflicting information on whether connections in V1 are strongly iso-linear [17] (tree shrew) or mostly isotropic [8] (cat). However, there is significant evidence from psychophysics [11,12] that lateral connections play a role in some forms of co-linear facilitation. It has also been demonstrated that connections are refined during development [3] and there are correlations in co-linear neuron spiking [18]. In summary, there is strong evidence for co-linear lateral connections, but also indications that other types of connections are present.

Previous work has shown that an important functional role of lateral connections is inhibitory, such as iso-orientation suppression [29]. However, the majority of connections we have considered here, particularly at longer distances (*r >*500 *μ*m), are believed to be excitatory [8,17]. Excitatory connections are known to be connected co-linearly and to be important for co-linear facilitation [11-13]. There is some evidence that V1 cells in some species may also be involved in border-ownership computation [30], however, since we are not aware of any characterisation of the type of connectivity necessary for these computations, we did not test the proposition here.

## Conclusions

We suggest that there may be a role for both anti-cocircular and co-circular lateral connections. Edges in natural scenes are preferentially co-circular (with a small but consistent bias). For tasks such as co-linear facilitation [12], excitatory co-linear lateral connections are necessary. This sort of facilitation is thought to be involved in contour integration [7]. However, from another point of view, it is precisely when input conflicts with what is expected, (in this case co-circularity), that it is providing novel and important information (indeed, it has been shown that high entropy areas of visual scenes are usually at the center of gaze [31]). From this point of view edges that are anti-cocircular are the most salient, and excitatory connections to facilitate their detection are a reasonable postulate. It is possible that the brain uses a combination of these strategies to both detect the unexpected while facilitating description of the more expected case (co-circularity). Our findings are consistent with such a bifurcated connection strategy.

An interesting experimental followup to this work would be to examine the lateral connectivity of animals reared under unusual rearing conditions (as done for orientation maps in [20]). Since these connections develop after eye opening it is likely that these rearing conditions would have an effect on lateral connectivity (indeed previous work has indicated that strabismus during development has an effect on connectivity [3]). This could provide further insight into the developmental mechanisms and statistics underlying lateral connections and the degree to which these connections are influenced by natural visual scenes during development.

## Competing interests

The authors declare that they have no competing interests.

## Authors' contributions

WHB acquired the experimental data. JJH, GJG designed the analysis. JJH performed the analysis. JJH, GJG, WHB wrote the paper. All authors read and approved the final manuscript.
